# Development and prospective validation of an artificial intelligence-based smartphone app for rapid intraoperative pituitary adenoma identification

**DOI:** 10.1038/s43856-024-00469-z

**Published:** 2024-03-13

**Authors:** Rabih Bou-Nassif, Anne S. Reiner, Matthew Pease, Tejus Bale, Marc A. Cohen, Marc Rosenblum, Viviane Tabar

**Affiliations:** 1 Department of Neurosurgery, Memorial Sloan Kettering Cancer Center, New York, NY USA; 2 Multidisciplinary Pituitary and Skull Base Tumor Center, Memorial Sloan Kettering Cancer Center, New York, NY USA; 3 Department of Epidemiology and Biostatistics, Memorial Sloan Kettering Cancer Center, New York, NY USA; 4 Department of Pathology, Memorial Sloan Kettering Cancer Center, New York, NY USA; 5 Department of Surgery, Head and Neck Service, Memorial Sloan Kettering Cancer Center, New York, NY USA

**Keywords:** Surgical oncology, CNS cancer, Cancer therapy, CNS cancer

## Abstract

**Background:**

Intraoperative pathology consultation plays a crucial role in tumor surgery. The ability to accurately and rapidly distinguish tumor from normal tissue can greatly impact intraoperative surgical oncology management. However, this is dependent on the availability of a specialized pathologist for a reliable diagnosis. We developed and prospectively validated an artificial intelligence-based smartphone app capable of differentiating between pituitary adenoma and normal pituitary gland using stimulated Raman histology, almost instantly.

**Methods:**

The study consisted of three parts. After data collection (part 1) and development of a deep learning-based smartphone app (part 2), we conducted a prospective study that included 40 consecutive patients with 194 samples to evaluate the app in real-time in a surgical setting (part 3). The smartphone app’s sensitivity, specificity, positive predictive value, and negative predictive value were evaluated by comparing the diagnosis rendered by the app to the ground-truth diagnosis set by a neuropathologist.

**Results:**

The app exhibits a sensitivity of 96.1% (95% CI: 89.9–99.0%), specificity of 92.7% (95% CI: 74–99.3%), positive predictive value of 98% (95% CI: 92.2–99.8%), and negative predictive value of 86.4% (95% CI: 66.2–96.8%). An external validation of the smartphone app on 40 different adenoma tumors and a total of 191 scanned SRH specimens from a public database shows a sensitivity of 93.7% (95% CI: 89.3–96.7%).

**Conclusions:**

The app can be readily expanded and repurposed to work on different types of tumors and optical images. Rapid recognition of normal versus tumor tissue during surgery may contribute to improved intraoperative surgical management and oncologic outcomes. In addition to the accelerated pathological assessments during surgery, this platform can be of great benefit in community hospitals and developing countries, where immediate access to a specialized pathologist during surgery is limited.

## Plain language summary

In tumor surgery, precise identification of abnormal tissue during surgical removal of the tumor is paramount. Traditional methods rely on the availability of specialized pathologists for a reliable diagnosis, which could be a limitation in many hospitals. Our study introduces a user-friendly smartphone app that quickly and precisely diagnoses pituitary tumors, powered by artificial intelligence (AI), which is the simulation of human intelligence in machines for tasks like learning, reasoning, problem-solving, and decision-making. Through data collection, app development, and validation, our findings demonstrate that the app can rapidly and accurately identify tumors in real-time. External validation further confirmed its effectiveness in detecting tumor tissue collected from a different source. This AI-driven app could contribute to elevating surgical precision, particularly in settings lacking immediate access to specialized pathologists.

## Abstract (web summary)

Bou-Nassif et al. develop a smartphone app using AI and stimulated Raman histology to differentiate pituitary adenomas from normal tissue in real-time during surgery. Prospective validation on 40 patients shows high sensitivity and specificity, with an external validation on 40 additional adenoma tumors.

## Introduction

Pituitary adenomas, accounting for 10–20% of all intracranial neoplasms, are the second most common intracranial neoplasm, with a 17% incidence in autopsy studies of the general population^1^. The majority of pituitary adenomas are benign and non-life threatening. While many tumors are initially observed, surgery is the first-line treatment for tumors demonstrating large size, fast growth, neurovascular compression, or hormone secretion, with the exception of prolactinomas^2^. One of the key principles in pituitary surgery is to maximally preserve normal gland tissue while resecting tumor. Resection of normal gland tissue can result in considerable hormonal deficiencies^3^. Distinguishing normal gland from abnormal tumor is especially relevant in surgery for functioning adenomas, where any residual disease is associated with higher recurrence rates and has the potential to shorten quantity and quality of life^4–6^ With gross total resection rates of only 75% for functioning adenomas, surgeons utilize a variety of tools, ranging from intraoperative MRI to 3D image guidance, to improve intraoperative resection rates^7,8^. Furthermore, a range of pathologies present as a sellar mass, and adenomas can present ectopically within the sphenoid sinus^9^. In the case of Cushing’s disease, surgeons often target very small tumors in the order of a few millimeters in diameters, and in the case of MRI-negative Cushing’s, the surgeon explores and biopsies the gland without the benefit of a definitive target identified on MRI imaging^10^. Hence, recognition of adenoma pathology is a valuable tool for intraoperative decision-making^11–15^. The Raman effect, discovered by Sir Chandrasekhara Venkata Raman in 1928, is a phenomenon in which light undergoes scattering by molecules, resulting in a change in frequency and wavelength, earning him the Nobel Prize in Physics in 1930. Stimulated Raman histology (SRH) is a method of 3D imaging that uses laser spectroscopy to analyze the chemical composition of samples. This method, described by Orringer et al.^11^, is an advancement of a new approach to stimulated Raman scattering (SRS) microscopy using high-repetition rate picosecond pulse trains, described by Freudiger et al.^12^. Unlike traditional hematoxylin & eosin (H&E) techniques that require freezing, sectioning and staining, SRH enables imaging of fresh tissue with minimal preparation, making it a valuable tool for studying raw specimens. SRH was shown to be comparable to traditional H&E, with the high contrast of the pink/purple color scheme similar to traditional stains, particularly in regard to highlighting nuclear features^13,14^.

Artificial intelligence (AI) is a field of computer science that trains computers to perform tasks by observing trends and rules in large datasets^15^. AI-based models have been shown to identify abnormalities in radiographic and pathologic images with accuracy approaching or exceeding expert physician specialists^16–18^. In pioneering work, Hollon et al. showed that SRH coupled with AI can play a major role in intraoperative cancer diagnosis^18^. They showed how SRH images obtained intraoperatively can be rapidly and accurately classified into different classes of brain tumors. Here we sought to develop a portable and specialized AI platform capable of accurately differentiating between pituitary adenoma and normal pituitary gland using SRH images during surgery. This platform, installed on a smartphone, for example, can then be used offline to capture a picture of an SRH image, instantly rendering a diagnosis. This would help guide surgical decision-making by providing confirmation of diagnosis and distinction between normal gland and abnormal tumor tissue, and improving the likelihood of a safe and complete tumor. This tool would have the advantage of being a binary classifier specialized in accurate tumor margin identification, in addition to the portability of the device on which it would be installed. This would also allow the repurposing of the model to different types of histology images, depending on the local availability of a particular technology or histology tools.

Expert intraoperative diagnostic interpretation for pituitary surgery is limited for several reasons. Most importantly, expert neuropathologists are unevenly distributed both within the United States and globally^19^. An estimated 40% of neuropathology fellowships in the US are vacant, leaving many neurosurgical centers without subspecialty expertise^20^. Additionally, when available, turnaround times for intraoperative frozen specimens may require 30 minutes or more to complete, with some surgeries requiring multiple samples during the same procedure to establish tumor-free margins. Lastly, many AI-based models face computational requirement hurdles that limit widespread adoption, especially in developing nations.

In this paper, to the best of our knowledge, we provide a detailed description of the development of a novel platform that uses a lightweight convolutional neural network (CNN) to accurately and instantly differentiate between pituitary adenoma and normal pituitary gland using SRH images. The use of lightweight CNN requires substantially less processing power and memory and makes it embeddable on a smartphone. We also describe the results of a prospective validation study conducted at our institution.

## Methods

### Patient selection and study design

**Fig. 1:**
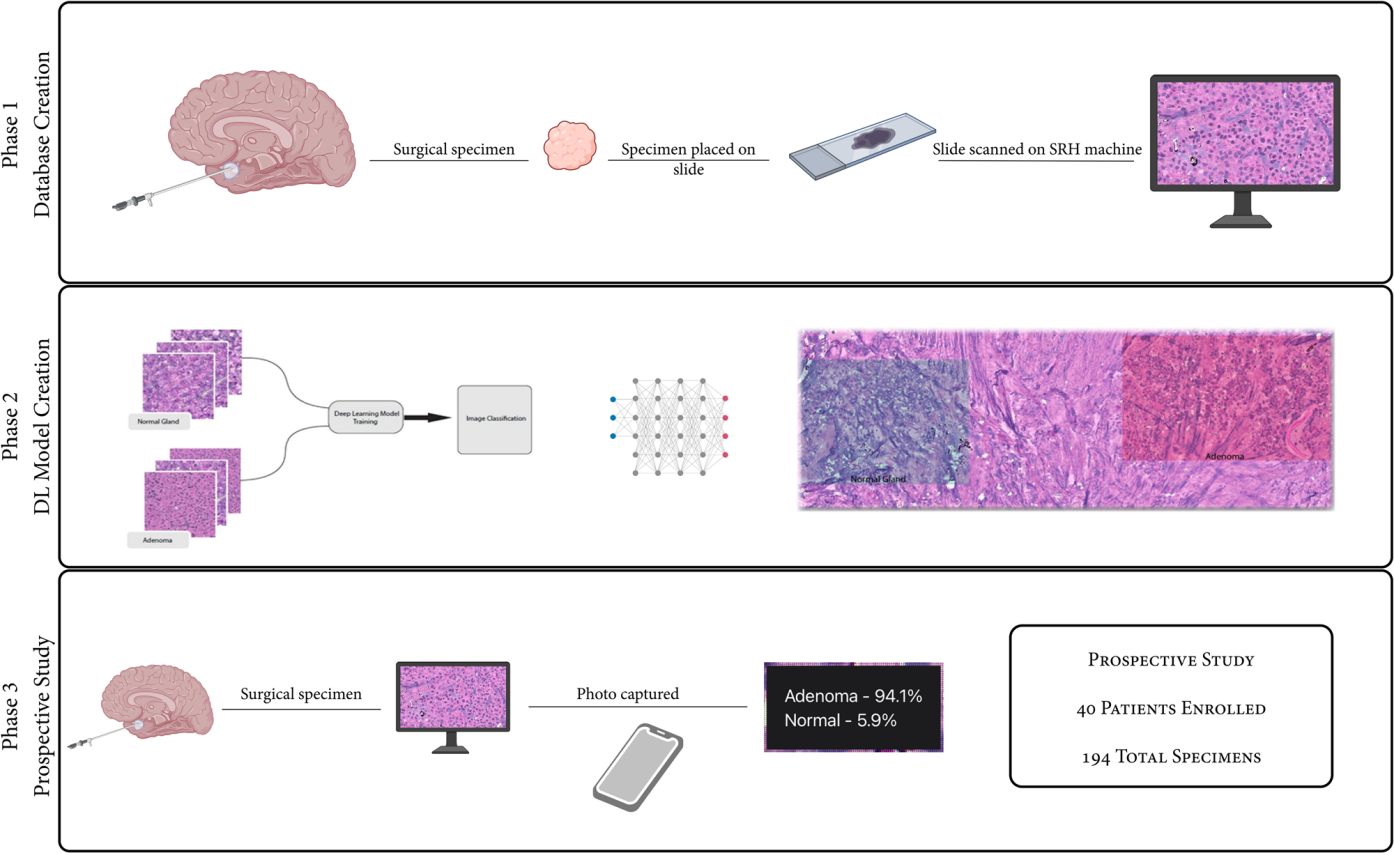
The Three Phases of the Study. Collection of the images and creation of datasets were performed in Phase 1. The deep learning models and the smartphone app were created in Phase 2. Phase 3 consisted of a prospective study to evaluate the app’s performance in a surgical setting.

We performed a prospective study developing and deploying an offline smartphone-based app using intraoperative SRH to distinguish between normal pituitary gland and pituitary adenoma. We included consecutive patients from August 2019 through December 2022 who underwent endoscopic transsphenoidal surgery to remove a pituitary adenoma at a single, tertiary referral center, by the same surgeon. We included all patients, regardless of tumor size, previous operation, or hormone secretory status, to more closely mimic a real-world environment. Data from this study were not used for clinical purposes. Our study was conducted in three phases: Phase 1—data collection; Phase 2—building a deep learning-based smartphone app; Phase 3—prospective study to evaluate the smartphone app (Fig. 1).

We followed the Standards for Reporting Diagnostic Accuracy Studies (STARD)^21^ and Transparent Reporting of a Multivariable Prediction Model for Individual Prognosis or Diagnosis (TRIPOD)^22^. The study was non-interventional and purely diagnostic in nature. Furthermore, the results from our app diagnosis tests did not alter patient care or influence clinical decision-making.

### Phase 1: Image and Dataset Creation

**Fig. 2:**
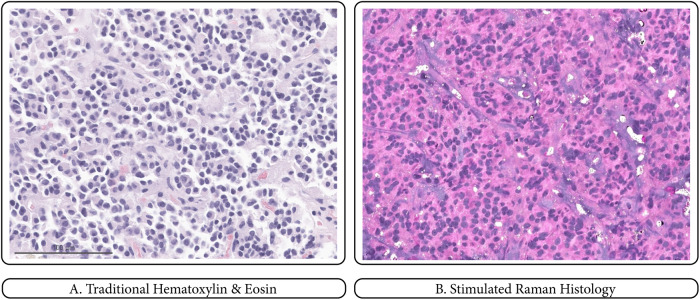
Comparison between Hematoxylin & Eosin and Stimulated Raman Histology. A traditional H&E slide is shown on the left **A** and an SRH image of the same specimen on the right **B**. The specimen was confirmed as a gonadotroph adenoma by formal pathological evaluation and immunohistochemical profile. Scale bar = 100 μm.

To train our deep learning model, we collected a large dataset of both normal pituitary gland and adenoma pathology images using intraoperative SRH from August 2019 to August 2021. SRH generates a color image by obtaining spectroscopic measurements at each pixel. (Fig. 2).

The image produced by SRH displays light absorption of CH2 molecular vibrations, found in lipids, and CH3 molecular vibrations, found in proteins and DNA. The system employs a high numerical aperture objective with 25× magnification and a 0.5 mm scan width. The automated stitching of multiple fields of view allows for the acquisition of larger areas up to 10 mm × 10 mm. The new SRS approach described by Freudiger et al. uses high-repetition rate picosecond pulse trains with low peak power^12^. This approach allows for more sensitive measurements than in previous reports and reduces low-frequency noise^17,18^.

If deemed safe by the attending neurosurgeon, a small intraoperative sample measuring approximately two to three millimeters in diameter is removed and placed on a translucent histology slide without any staining, processing or sectioning. The specimens’ total surface area ranged from 2 to 20 mm^2^ when smeared on the slide. We performed SRH imaging on each slide using the NIO Laser imaging System by Invenio Imaging Inc (Santa Clara, CA). Typically, we scanned an area of 2 mm × 2 mm first to have an SRH image displayed quickly, but often expanded image size to cover the whole specimen if applicable. The processing and scanning time for a 2 mm × 2 mm scan was 2 min. After scanning was complete, we fixed the scanned specimen in formalin and a board-certified neuropathologist provided a formal diagnosis. The neuropathologists’ final permanent diagnosis on H&E sections (and any additional immunohistochemistry ordered by the pathologist) performed on the same specimen used for intraoperative SRH diagnosis served as the ground-truth label.

To increase the number of normal pituitary gland samples, we collected fresh pituitary glands from the Last Wish Program, a rapid autopsy research program that enables patients at the end of their life to donate their organs for research at Memorial Sloan Kettering Cancer Center. Immediately postmortem, we collected normal pituitary glands from whole body or tissue donations. The specimens were used fresh without any processing, as it is usually done for specimens collected and scanned during surgery. They were sliced and scanned using the same SRH technique described above.

After acquiring whole slide SRH images, pre-processing was performed to develop a large database for model building and to adapt the training image size to the corresponding model. The images were cropped and cleaned of non-diagnostic areas using a Numpy array slicing method^23^, in Python 3.8. The images were sliced into 299 × 299 pixels patches. The sliding step for patch creation was 299 pixels horizontally and vertically, resulting in no overlap between patches. The no-overlap method was preferred in order to create completely distinct patches for model training, thereby reducing internal model validation bias during the training. All SRH image patches were then manually checked to confirm labels, and any regions without visible nuclei were discarded.

### Phase 2: CNN model creation and smartphone app development

Using our database of pathology images, we built our CNN model using CoreML, which is Apple’s proprietary machine learning framework that is designed to interface with Apple smartphones. This model has the advantage of perfect compatibility with iOS interfaces. This allowed us to install a lightweight CNN into an iOS smartphone. We created our CoreML model in a Swift framework using Xcode 12.0. We used common data augmentation techniques including rotation and flipping to increase training data. The model’s performance was evaluated using hold-out test dataset with an 80-20 split of the total number of pathology images.

We used a 32 GPU core device with a 16-core Neural Engine Apple Silicon M1 Max for model building with 64GB of unified memory.

Our smartphone app was designed to allow users to take a picture of the SRH screen, implement the deep learning model, and report a diagnostic certainty in a near-instantaneous manner. We installed our smartphone app on an iOS 14.1 device (Apple Inc., Cupertino, California, United States) with a dual 12MP wide camera. Similar to the first model, we tested the model performance with an 80-20 split from the dataset of pathology images.

### Phase 3: Prospective Study

**Fig. 3:**
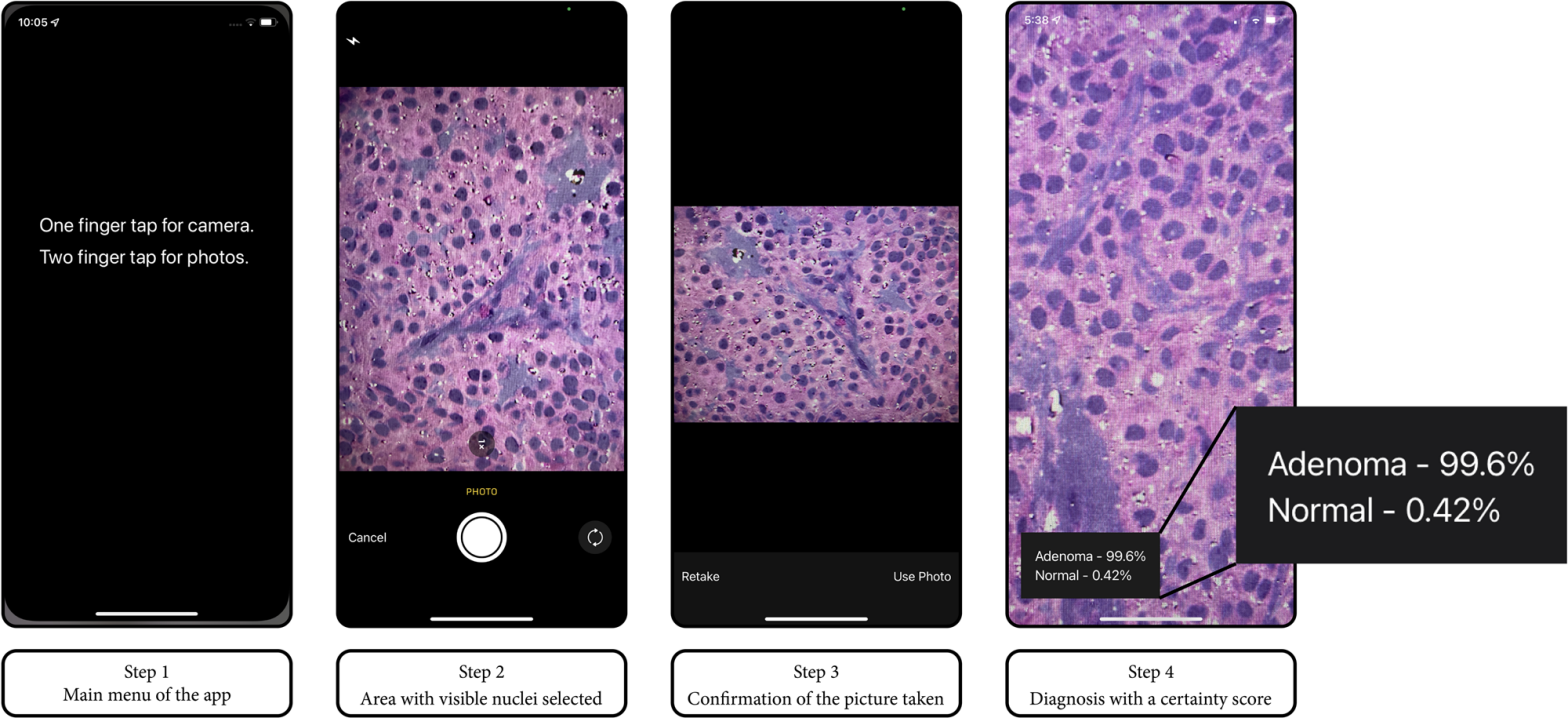
The Four-Step Workflow Ivolved in Utilizing the Application. Step 1 shows the main menu of the app. In step 2, the user focuses with a tap on the screen on an area with visible nuclei. In step 3, the user has the option to confirm or to retake a new picture. In step 4, the app renders a diagnosis with a certainty score.

To test our app, we performed a prospective, blinded study to evaluate the diagnostic accuracy of consecutive suspected pituitary adenomas from October 2021 through December 2022. The app operator was not provided with any information or feedback from a pathologist, thus ensuring that the operator’s observations were independent and unbiased. We scanned the specimen using SRH as described above. The operator visually scanned the image for an area with nuclei and took a landscape picture using the smartphone app, with the device at approximately 20 cm from SRH image (Fig. 3). Similar to the data collection in Phase 1, the SRH tissue slide was then fixed in formalin and sent to neuropathology for the permanent diagnosis, which was considered the ground-truth for that specific specimen.

For our study, we considered a certainty score above 70% as diagnostic. We chose this cutoff based on a survey reported by Bracamonte et al. among board-certified pathologists that showed that a “consistent with” diagnosis by a pathologist corresponded to a certainty of approximately 70%^24^. Lower scores were considered non diagnostic, and we repeated the app evaluation. In all cases, improving the focus and picture quality improved certainty above 70%.

We further tested our model by running an analysis on the raw images directly, without using a smartphone or camera.

### External validation

For greater confidence in the app, we also performed an external validation using OpenSRH (https://opensrh.mlins.org) which is the only publicly available dataset of clinical SRH images sourced from different brain tumors, including pituitary adenomas^25^. We downloaded 191 SRH images originating from 40 cases of pituitary adenomas, then used our smartphone app for diagnosis based on the entire SRH image with the same technique used in our Phase 3 study.

### Statistical methods

To assess model performance in discriminating between normal pituitary and abnormal gland, we reported the following metrics: accuracy, F1 score, precision, and recall. The F1 score is a commonly used metric in binary classification tasks and is defined as the harmonic mean of precision and recall. Precision is the ratio of true positive predictions to the total number of positive predictions, while recall is the ratio of true positive predictions to the total number of actual positive samples. The F1 score provides a balance between precision and recall, and it is particularly useful when the class distribution is imbalanced. A high F1 score indicates that the model has a good balance of precision and recall, meaning that it can accurately identify both positive and negative samples. To assess app performance discriminating between normal pituitary gland and pituitary adenoma, we reported the following metrics: sensitivity, specificity, positive predictive value (PPV), and negative predictive value (NPV). Given that our data was clustered, with some patients having multiple pathology specimens per surgery, we estimated 95% confidence intervals (CIs) accounting for intra-patient variation using the Taylor series method estimates for variance among clusters.

We displayed the histogram of the certainty for each prediction when classified by ground truth (i.e., normal gland or adenoma) to demonstrate the degree of certainty for each class. The floor for our certainty histograms is 70% as we repeated any sample with a reported certainty below 70%. The certainty score distribution was compared by ground truth using the t-approximation of the Wilcoxon Two-Sample test. We performed all analysis using SAS version 9.4 (The SAS Institute; Cary, NC) and R version 4.2.2 (The R Foundation for Statistical Computing; Vienna, Austria).

### Ethical considerations

Our study was determined to be exempt from IRB review at Memorial Sloan Kettering Cancer Center under the regulations governing research with human subjects (45 CFR 46.104). Therefore, a specific written consent was not required for this biospecimen diagnostic study, and no clinical decisions were based on its results.

## Results

Dataset creation for model training (Phase 1) went from August 2019 to September 2021. It included 56 cases where adenoma tissue was sampled and 25 cases where normal pituitary gland was sampled. We supplemented our normal gland dataset with five normal whole pituitary glands collected from fresh autopsies, described in the methods section.

After splitting into training and testing datasets, and the preprocessing described in the methods section, a total of 32,051 unique training images (16,694 adenoma images and 15,357 normal pituitary gland images). The testing dataset consisted of 8,013 images (4174 adenoma images and 3839 normal pituitary gland images). The CoreML model had a 95.2% accuracy, precision of 93% and recall of 93%, with an F1 score of 0.93.

### Prospective Study Results (Phase 3)

**Fig. 4:**
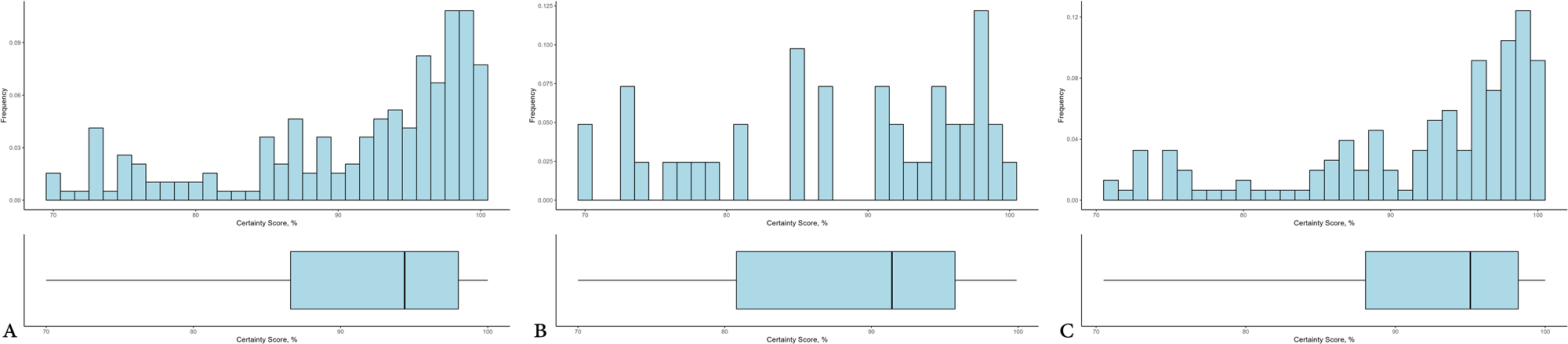
Distribution of the Smartphone App Certainty Score. **A** Distribution of the smartphone app certainty score across all tests. **B** Distribution of the smartphone app certainty score across all tests where ground truth was normal pituitary gland. **C** Distribution of the smartphone app certainty score across all tests where ground truth was adenoma.

The prospective study for the evaluation of the performance of the app in a surgical setting included 40 consecutive patients from October 2021 to December 2022, without any exclusion. A total of 194 samples were tested. A neuropathologist evaluated each sample to determine ground truth by providing permanent section diagnosis on the same specimen. The results of the app were compared to ground truth and the following performance measures were obtained: sensitivity was 96.1% (95% CI: 89.9–99%), specificity was 92.7% (95% CI: 74–99.3%), PPV was 98% (95% CI: 92.2–99.8%), and NPV was 86.4% (95% CI: 66.2–96.8%). Furthermore, the certainty score of the app across all tests (Fig. 4A) was analyzed and the following distribution statistics were obtained: N was 194, minimum was 70%, 25th percentile was 86.6%, median was 94.4%, mean was 91.1%, 75th percentile was 98%, and maximum was 100%.

Supplementary Data 1 displays the study patient list with the intraoperative and final pathology diagnosis, the clinical presentation, and the app errors.

The certainty score of the App across all tests where the ground truth was normal pituitary gland (Fig. 4B) was analyzed. The following distribution statistics were obtained: *N* = 41, minimum was 70%, 25th percentile was 80.8%, median was 91.4%, mean was 88%, 75th percentile was 95.7% and maximum was 99.9%.

The certainty score of the smartphone application across all tests where the ground truth was tumor tissue (Fig. 4C) rendered the following distribution statistics: N = 153, minimum was 70.5%, 25th percentile was 88%, median was 95%, mean was 91.9%, 75th percentile was 98.2% and maximum was 100%.

The certainty score was statistically significantly higher when the ground truth was tumor tissue (*p* = 0.0089).

The additional testing done on the transferred SRH images yielded a 92.3% accuracy (95% CI: 86.2–96.3%), 93.5% sensitivity (95% CI: 86.4–97.5%), 87.8% specificity (95% CI: 72.5–96.3%) with a PPV of 96.6% (95% CI: 90.7–99.3%), and NPV was 78.3% (95% CI: 57.6–92.0%).

### External validation

For greater confidence in the app, we tested it on entire SRH images retrieved from a publicly available database from different institutions. We downloaded 191 SRH images from 40 different adenoma cases from OpenSRH and interrogated the app for diagnosis. The sensitivity of our smartphone app accurately diagnosing adenoma was 93.7% (95% CI: 89.3–96.7%). The specificity couldn’t be determined because the dataset does not contain normal pituitary gland images.

## Discussion

We demonstrate that a deep learning, smartphone app using stimulated Raman histology can successfully and very rapidly differentiate between normal pituitary gland and adenoma. Pathologists play a vital role in the diagnosis and management of diseases by analyzing tissue samples and identifying abnormalities at the cellular level. Despite advances in molecular pathology, there remains a substantial need for rapid intraoperative diagnosis to guide surgical decision making. However, the global demand for specialized pathologists often exceeds the supply, resulting in a shortage at many centers with neurosurgical expertise^25^. This shortage of pathologists is particularly acute in certain regions, such as sub-Saharan Africa, where the relative number of pathologists is one tenth that of most developed nations^26^. Between 2007 and 2017, in the United States, the number of active pathologists decreased by 17% and the diagnostic workload per pathologist increased by 41%^27^. This shortage can lead to delays in diagnosis as samples may need to be sent to distant laboratories for analysis. One possible solution for acute shortages is utilizing AI-based technologies to extend expert physician reach^25^. We propose one solution, utilizing a customized CNN model to achieve intraoperative diagnostic pathology results that are comparable to historical norms. Our smartphone app, prospectively validated in a study, could be rapidly deployed in resource-limited regions without limited pathological expertise.

Hollon et al. demonstrated that a combination of SRH and deep convolutional neural networks is non-inferior to traditional pathologist-based diagnosis and much faster^18^. They showed how intraoperative cancer diagnosis can be streamlined, creating a complementary pathway for tissue diagnosis that is independent of a traditional pathology laboratory.

Our workflow, which obtained high certainty scores within minutes, is a substantial added tool to current workflows for intraoperative frozen section with H&E staining and interpretation from expert pathologists. Timely intraoperative pathology can enhance surgical effectiveness and contribute to informed surgical decision-making, with little downtime. For transsphenoidal pituitary surgery, difficulty differentiating between normal pituitary gland and pituitary adenoma in situations where access to neuropathologists is lacking can lead to undue damage to normal gland or incomplete resection of viable tumor. This could potentially be prevented by taking out a millimetric fragment of tissue, analyzing it almost instantly to know if it is normal pituitary gland indicating that the resection in that area is sufficient. On the other hand, if this tiny specimen turned out to be tumor, it would indicate a need to pursuing the resection. Furthermore, extending surgical time while waiting for pathology results, sometimes several times during a single surgery, may increase risks for infections or other intraoperative complications. It is estimated that every 30 minutes of increased surgical time increases the likelihood of a complication by 14%, in addition to increased costs^28,29^.

Despite obtaining rapid intraoperative pathology results within minutes, our workflow does not sacrifice quality. Our F1 score, a common performance marker for binary classification tasks, demonstrates that our model works well. Similarly, we obtained a high degree of confidence, typically exceeding 90%. We achieved this performance with minimal performance loss when using machine learning frameworks designed to work on smartphones (CoreML). Our data demonstrate that a high level of accuracy and rapidity can be achieved with minimal technological support, with the potential to extend pathology expertise with easy-to-use technologies.

External validation on a new dataset, created at different institutions on different SRH machines confirmed the high sensitivity of the smartphone app in detecting pituitary adenoma, even when changing the work environment.

Our work has several limitations. First, this is a single center study, which can introduce bias. Future work should include multicenter validation of the platform. Even though we performed an external validation on an adenoma dataset, it is still limited by the unavailability of external datasets of normal pituitary gland images. Second, the deep learning model presented here is trained to specifically distinguish between normal pituitary gland and pituitary adenoma. Future models can be trained to identify a large number of different sellar pathologies and other tumors. Additionally, further work would have to validate the value of this platform in cases where a prior resection was performed, or radiation was administered.

Furthermore, factors like room illumination, monitor settings, and mobile phone camera settings could introduce perturbations that might affect the accuracy of detection. Nevertheless, the additional testing we did on the images without going through the smartphone or camera shows comparable results to the smartphone app. In addition, the prospective study done in Phase 3 has demonstrated the effectiveness of our technique in real-world conditions, further validating its practicality and robustness.

Another limitation of our platform is its current specificity to SRH images, which may restrict its use due to financial or proprietary considerations. Nonetheless, the versatility of our platform allows for its potential adaptation to different optical images, such as SRS images, and even classic H&E images. In the latter case, an affordable adapter such as described by Liu et al. could be attached to the smartphone’s camera to capture an image shown through a traditional microscope^30^. This would be particularly relevant in developing countries where the cost of access to H&E preparations is substantially lower than the cost of Raman-based technologies, and where there is a dire need for specialized pathologists. The use of a modified version of our platform could help address this need by providing an accessible and affordable solution for diagnosing a variety of pathologies, particularly in settings with limited access to specialized resources. Incorporating our platform in the surgical workflow at centers with limited access to high-quality intraoperative diagnosis could potentially improve maximal surgical resection of tumors. This, in turn, may reduce the need for repeat surgeries and enhance oncologic care and patient-reported outcomes.

### Supplementary information

Description of Additional Supplementary Files

Supplementary Data 1

Supplementary Data 2

## Data Availability

Source data for the figures can be accessed as Supplementary Data 2. SRH image datasets are available from the corresponding author upon reasonable request.

## Code availability

Code to preprocess the images, train the deep-learning models, and create the mobile app are available on our GitHub repository^31^.
