# Supplementary material for: Development and prospective validation of an artificial intelligence-based smartphone app for rapid intraoperative pituitary adenoma identification
Source: Commun Med (Lond). 2024 Mar 13;4:45. doi: 10.1038/s43856-024-00469-z (PMC10937994; doi:10.1038/s43856-024-00469-z)
Supplement: Supplementary file 1 — Description of Additional Supplementary Files [file 43856_2024_469_MOESM1_ESM.pdf]

Description of Additional Supplementary Files

File Name: Supplementary Data 1
Description: The prospective observational study patient list with the intraoperative and final pathology diagnosis, the clinical presentation, and the app errors. PitNET, pituitary neuroendocrine tumor; SF1, Steroidogenic factor 1; NF, non-functioning.

File Name: Supplementary Data 2
Description: Source data for Figure 4.
